# MCMC algorithm based on Markov random field in image segmentation

**DOI:** 10.1371/journal.pone.0296031

**Published:** 2024-02-22

**Authors:** Huazhe Wang, Li Ma

**Affiliations:** 1 College of Computer Engineering, Shangqiu Polytechnic, Shangqiu, China; 2 Soft Vocational Technology Institute, Shangqiu Polytechnic, Shangqiu, China; Nanjing University of Information Science and Technology, CHINA

## Abstract

In the realm of digital image applications, image processing technology occupies a pivotal position, with image segmentation serving as a foundational component. As the digital image application domain expands across industries, the conventional segmentation techniques increasingly challenge to cater to modern demands. To address this gap, this paper introduces an MCMC-based image segmentation algorithm based on the Markov Random Field (MRF) model, marking a significant stride in the field. The novelty of this research lies in its method that capitalizes on domain information in pixel space, amplifying the local segmentation precision of image segmentation algorithms. Further innovation is manifested in the development of an adaptive segmentation image denoising algorithm based on MCMC sampling. This algorithm not only elevates image segmentation outcomes, but also proficiently denoises the image. In the experimental results, MRF-MCMC achieves better segmentation performance, with an average segmentation accuracy of 94.26% in Lena images, significantly superior to other common image segmentation algorithms. In addition, the study proposes that the denoising model outperforms other algorithms in peak signal-to-noise ratio and structural similarity in environments with noise standard deviations of 15, 25, and 50. In essence, these experimental findings affirm the efficacy of this study, opening avenues for refining digital image segmentation methodologies.

## 1 Introduction

Stepping into the 21st century, the development of computer science and technology is changing day by day, in which the processing and analysis of digital images have formed a unique scientific system [1]. In the processing and analysis of digital images, image segmentation (IS) is a key part [2]. However, effective image segmentation is not easy to realize in adaptive image processing due to insufficient a priori information and imaging noise [3]. In addition, traditional image segmentation methods usually use manual threshold selection or region growing-based methods, which require a lot of manual intervention and are prone to errors and omissions [4]. In this context, the research innovatively proposes a new image segmentation algorithm based on Markov Chain Monte Carlo (MCMC), which is optimized for Markov Random Field (MRF). This method represents the integrals as distributional expectations by MCMC. And it estimates the complex integrals in image segmentation processing by sampling the sample points that follow the distribution. In addition, to address the imaging noise, an adaptive window similarity block search algorithm based on MCMC sampling is used to optimize the non-local denoising algorithm [5]. The better image segmentation results can be obtained by converting images containing noise or noises into purer sequences. This processing and optimization technique allows the algorithm to perform exceptionally well when managing intricate image segmentation tasks with substantial benefits in minimizing manual intervention and errors. This study mainly consists of five parts. The first part is an overview of the research. The second part is a summary of relevant work at home and abroad. The third part is divided into two sections. The first section introduces the image segmentation algorithm of MRF-MCMC, and the second section introduces the adaptive segmentation image denoising algorithm based on MCMC sampling. The fourth part is the experimental verification of the proposed method in the research. The fifth section is a discussion of the research methodology and its innovativeness and effectiveness in applications such as computer vision and image processing. And the sixth section summarizes the research and looks forward to future research. The research introduces the basic principle and implementation method of MRF-MCMC, including image preprocessing, feature extraction, target segmentation, and other steps. And a comparison of image segmentation and denoising algorithms is designed to verify the application effect of the MRF-MCMC algorithm, aiming to provide new references for digital image analysis and processing.

## 2 Related works

In digital image processing, image segmentation is a very important topic. It is the foundation of image processing and understanding. At present, image segmentation technology has been widely applied in security monitoring, remote sensing imaging, and clinical medicine. Many scholars have conducted extensive research on image segmentation technology and its optimization methods. Luo S et al. proposed a new convex representation calculation method in image segmentation processing. And they demonstrated that shape convexity was equivalent to quadratic constraints of related index functions. This method was studied to improve probability-based models for extracting convex prior targets from images. The superiority of this method had been verified through image segmentation experiments [6]. Jiang Zhe et al. found that although deep learning had achieved certain results in image segmentation applications, its effectiveness in real-world applications was not ideal due to the lack of high-quality training labels. Therefore, this paper proposed a weak supervised learning framework. This learning framework could simultaneously update deep learning model parameters and infer hidden label positions. By improving the training labels in this way, it aimed to increase the effectiveness of image segmentation in real-world applications. The tests on real datasets had found that this method had better classification accuracy than other similar methods [7]. Kaushal C and other scholars proposed an image segmentation technology based on Firefly algorithm, which simulated mathematics and innovative technologies to solve the global nonlinear and real life problems. This technique could be used to segment breast cancer images, regardless of the type or mode of the image. The effectiveness of this technology was verified by comparing the obtained results with the most advanced existing technology. The experimental results denoted that the proposed image segmentation method based on the Firefly algorithm was efficient in processing medical images, such as cancer cell nucleus detection, blood vessel segmentation, organ or tissue structure research. And it was comparable to the existing advanced technology [8]. Kumar A et al. proposed an improved image segmentation method for crop image analysis. When the traditional minimum cross entropy method was used for multi-level threshold segmentation, the computational complexity increased. The recursive minimum cross entropy was used to solve the computational complexity. And the Cuckoo search based on Levy flight was used to find the optimal threshold of the objective function. The accuracy of this method was tested on 10 crop images. The experimental outcomes denoted that the proposed method provided the most promising results and improved accuracy [9]. Yan X and Weng proposed an optimized fast image segmentation model based on local pre-fitting images. This model combined the regional features of local pre-fitted images and digital images, which not only accurately segmented images with blurred boundaries, but also had performance in resisting large noise. In addition, the study calculated the pre-fitted function before the curve evolution, which significantly reduced the computational complexity of the model. The research findings indicated that the image segmentation efficiency of this model was superior to other comparative models, and it had good parameter robustness [10].

Usually, image denoising is considered as a part of image restoration and a preprocessing step before image segmentation. Image denoising can effectively improve the efficiency of image segmentation and image processing. Therefore, a large number of scholars have conducted research on image denoising and proposed different denoising methods. Ma R et al. proposed a new proportional integral and differential attention network to address over-fitting and low performance of deep convolutional neural networks in real-world noisy image denoising. Research guided the learning framework to adaptively update by stacking attention modules and utilizing second-order statistical feature correlation. The effectiveness of the proposed method was verified in the experimental results on the dataset [11]. Han L et al. conducted research on deburring of mechanical surfaces. To obtain more accurate burr images, a new burr model was constructed and a real dataset was established for image denoising. To improve the denoising effect, an online denoising algorithm was also proposed. This algorithm had good adaptability to images with inherent noise. In the experimental results, this algorithm outperformed other traditional and deep learning algorithms, which had the best denoising effect [12]. Ghorbanzadeh O et al. proposed an accurate edge extraction method based on image analysis to avoid the impact of noise imaging on image analysis. The study excluded image edge noise through visual measurement-based image metric characteristics, thereby achieving the detection of the wear of sliding parts of trains during high-speed operation. The experiment outcomes expressed that this algorithm could accurately detect pixel edges in images, and its detection accuracy for wear of train sliding parts was much higher than other methods [13].

In summary, some scholars have improved the training labels by constructing a weakly supervised learning framework to increase the effectiveness of image segmentation for real-world applications. Some scholars have computed the prefitting function, through which the computational amount of the image segmentation model has been significantly reduced. But there are few studies that directly improve the accuracy of segmented images [14]. And the existing segmentation accuracy has been unable to meet the needs of modern society [15]. For this reason, the research innovatively proposes the MCMC image segmentation algorithm based on MRF model. In addition, the study also combines the advantages of different image denoising algorithms and develops an adaptive segmentation image denoising algorithm based on MCMC sampling. The research aims to provide more effective image segmentation techniques in the field of image processing.

## 3 Application of MCMC algorithm based on MRF in image segmentation processing

MRF is a probabilistic model that accurately models the interrelationships between components in a multidimensional dataset. And it has a wide range of applications in various fields, such as computer vision, machine learning, and image processing. MRF can provide an effective way of comprehensively understanding and parsing the data, taking into account a certain amount of contextual information. In this research, the MCMC algorithm is optimized based on MRF to improve the effectiveness and accuracy of image segmentation. The study also introduces an adaptive window similar block search algorithm based on MCMC sampling to optimize the non-local denoising algorithm. This optimized non-local denoising algorithm is used as a pre-processing step aimed at enhancing the accuracy and robustness of image segmentation by reducing noise. In this way, image denoising and segmentation are combined with each other and work together to form a unified and synergistic image processing framework. This not only improves the effectiveness of each individual step, but also enables a better image understanding and analysis through their interaction.

### 3.1 Image segmentation algorithm based on MRF for MCMC

**Fig 1:**
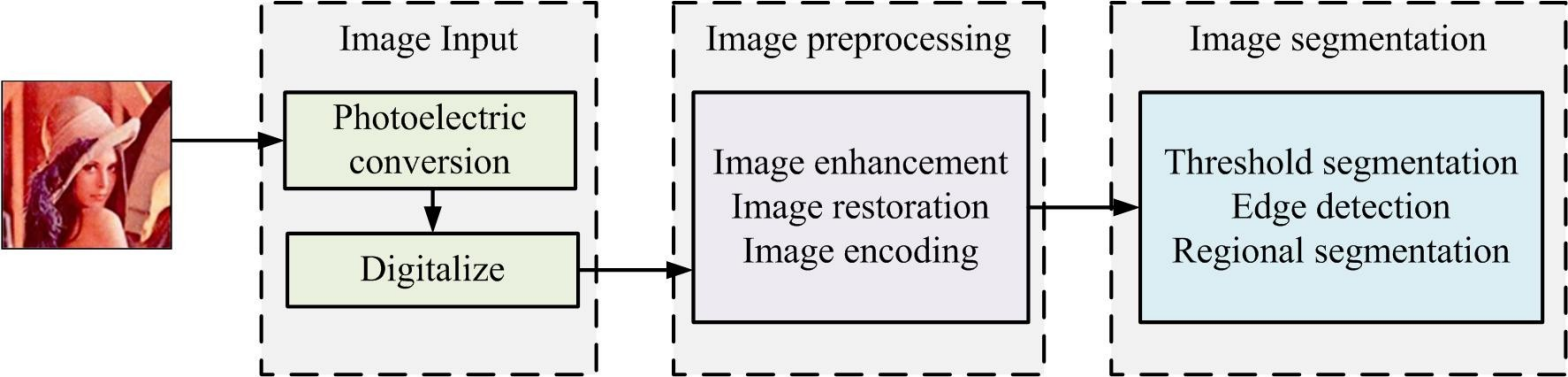
Image processing.

Image segmentation is a key step in image processing and is used to divide an image into multiple regions for subsequent analysis. With the advancement of computer technology, the application scope of image segmentation is expanding. For example, in medical images, anatomical structures can be extracted using threshold segmentation. In remote sensing pictures, target regions are detected by region growing algorithms. In industrial inspection, edge detection algorithms can recognize the contours of metal objects [16]. The complete image processing steps are shown in Fig 1.

As shown in Fig 1, the image processing is mainly divided into three parts, including image input, preprocessing, and segmentation [17]. Image processing covers photo conversion and digitization of image input, preprocessing, and image segmentation using techniques such as thresholding, edge detection, and regioning. After processing, depending on the objective, feature extraction or image recognition is performed. Then the processed image is analyzed and understood and finally interpreted. However, traditional image segmentation requires a large amount of a priori information or it is not effective [18]. For this reason, an image segmentation algorithm is proposed based on MRF to reduce the dependence on a priori knowledge. The MRF model can describe the local areas of an image. The interaction between local pixels is represented by low-order MRF, and the set of low-order MRF represents the energy function of the entire image [19]. It has been proved that only when the random field (RF) is the Gibbs distribution of the neighborhood system ∂, the RF is the MRF. The equivalent form of Gibbs distribution and MRF is shown in Eq (1) [20].


$$P(X=x)=\frac{1}{Z}exp[-\frac{1}{T}\sum_{c\in C}V_{c}(x_{s}|x_{r})]$$ (1)

In Eq (1), *Z* means the normalization constant. *T* represents the temperature constant. *C* denotes a set of neighboring systems containing functional groups. *S* is a finite set of points at the location. *X* means the RF at *s*∈*S*. *x*_*s*_ is the hidden state random variable on the RF. *V*_*c*_ expresses the potential formula on group *C*. After the prior distribution and likelihood function of image samples are obtained, the prior knowledge of the image can be transformed into a distribution model through Bayesian marking, to solve the uncertainty of MRF description. In this study, image segmentation is performed with the Maximum A Posterior (MAP). MAP is an estimation method based on Bayesian statistics, which is used to find the most likely values of model parameters under given observation data [21]. The optimal RF is calculated on the basis of MAP to maximize the posterior probability distribution of the RF and minimize the pixel classification error probability, as shown in Eq (2) [22].


$$X=argmaxP(X|Y)=argmax\frac{P(X|Y)P(X)}{P(Y)}$$ (2)

**Fig 2:**
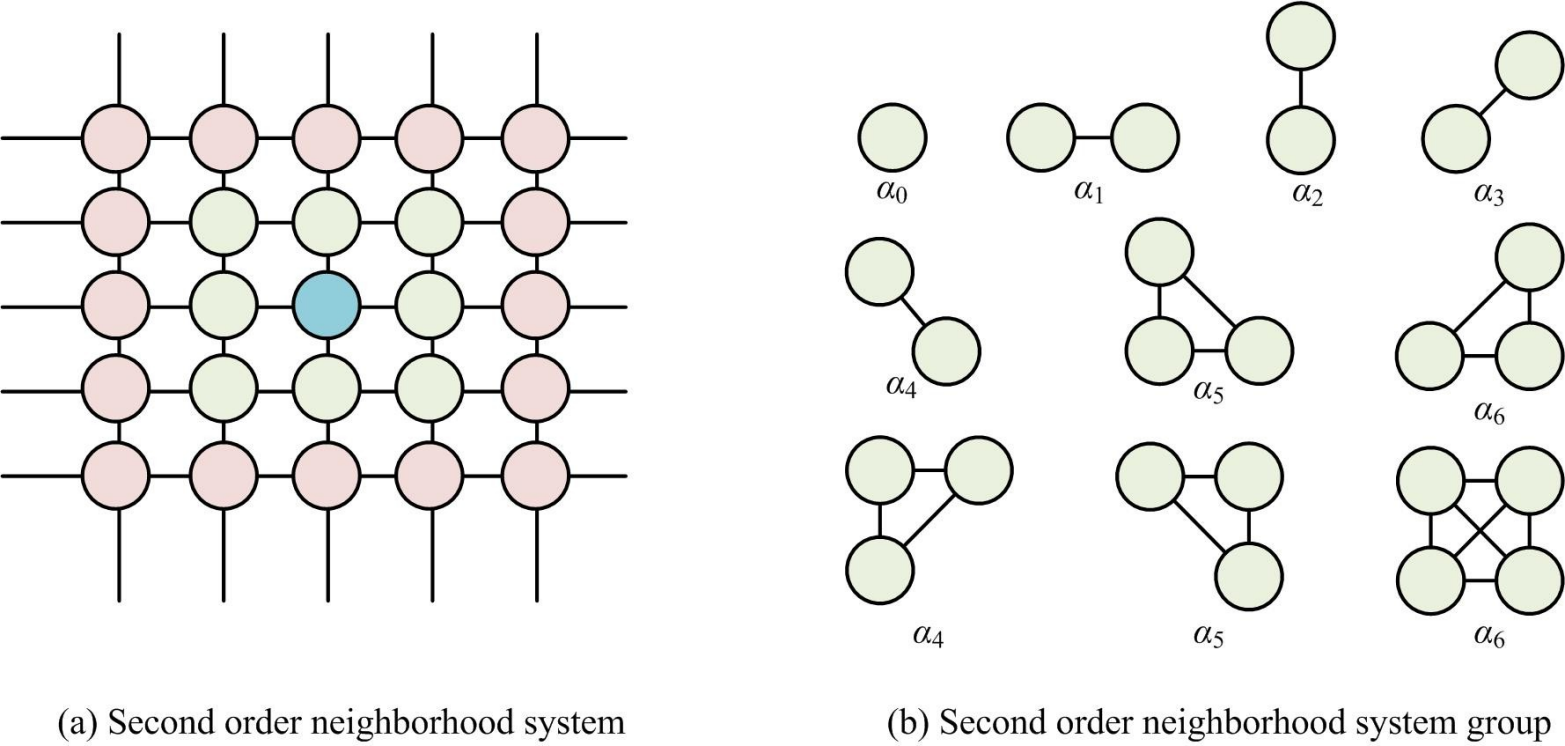
Schematic diagram of a second-order neighborhood system and its atomic groups. (a) Second order neighborhood system and (b) Second order neighborhood system group.

In Formula (2), *X* and *Y* represent two different RF describing images, respectively. *X* is used to describe the local correlation of pixels, and *X* = {*x*_*s*_|*s*∈*S*}. *Y* is the observation field used to represent the distribution of observation data, and *Y* = {*y*_*s*_|*s*∈*S*}. The label field prior model can be represented by a Multi Level Regression (MLL) model. The MLL model is mainly used to analyze the relationship between a binary response variable and one or more predictive variables, while considering the hierarchical or grouping structure of the data [23]. In this model, it not only estimates fixed effects, but also random effects, i.e. variations between different groups or levels. This study uses a second-order neighborhood system (SONS) as a prior model for labeling fields. The SONS and its atomic clusters are shown in Fig 2.

Fig 2(A) is a schematic diagram of a SONS, where the blue dots represent any pixel, and the blue dots and surrounding green dots get together to form the SONS [24]. Fig 2(B) is the atomic clusters in a SONS, where the central pixel and the pixels in the SONS form different atomic clusters [25]. Then, according to the Bayesian criterion, the maximum posterior probability is transformed into the energy function. For the feature field model, the grayscale attribute of the image is described by the Gaussian function, as shown in Eq (3).


$$P(y_{l}|x_{l})=\frac{1}{\sqrt{2\pi }\sigma_{x_{l}}}exp\{-\frac{{\left(y_{l}-\mu_{x_{l}}\right)}^{2}}{2\sigma_{x_{l}}^{2}}\}$$ (3)

In Eq (3), $\mu_{x_{l}}$ denotes the average pixel grayscale value of each label *x*_*l*_. $\sigma_{x_{l}}$ means the standard deviation of each label *x*_*l*_. To avoid situations where the initial values of parameters are sensitive, the Monte Carlo (MC) is applied to set the initial state. In parameter inference of iterative algorithms, the random vector reflects the situation of the observation vector. In Bayesian estimation, by simplifying the complete dataset model *f*(*y*|*θ*), which is recorded as *y*_*obs*_ and the rest as *y*_*mis*_, the analytical form of posterior probability is found. *y* can be seen as the combination of *y*_*obs*_ and *y*_*mis*_, where *π*(*θ*,*y*_*mis*_) and $f(y_{mis},y_{obs}|\theta )f_{0}(\theta )$ are proportional in the joint PD. The PD of the observed data is shown in Eq (4).


$$(\theta |y_{obs})=\pi (\theta )=\int \pi (\theta ,y_{mis})dy_{mis}$$ (4)

By obtaining MC samples from the joint PD, the histogram of sample parameters can be utilized as an approximate PD of observation data. Due to the PD requiring integration of high-dimensional functions, a large amount of computation limits Bayesian inference [26]. The research uses MCMC algorithm to express the integral as the distribution expectation, and estimates the complex integral in image segmentation processing by sampling the sample points that obey the distribution. The basic principle of MCMC is to sample a stable distribution MC to obtain a large number of samples, and complete the required statistical inference through these samples [27]. Taking the basic method of MCMC as an example, when solving a problem, it is necessary to determine the random variable *x* and calculate the statistic *g*(*x*), so that the mathematical expectation ∫*g*(*x*)*f*(*x*)*dx* of the statistic is equal to the target value. *f*(*x*) represents the density function of a random variable. The average observed expression of MCMC is shown in Eq (5).


$$A=\frac{\int A(x)\pi (x)dx}{\int \pi (x)dx}$$ (5)

**Fig 3:**
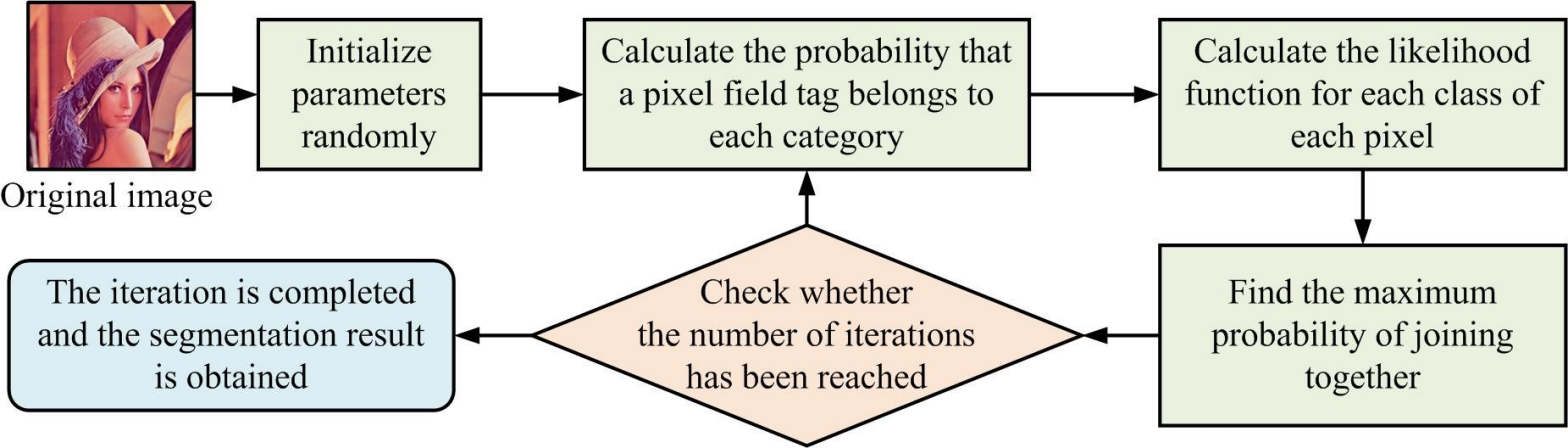
Markov random field-Markov Chain Monte Carlo image segmentation algorithm flow.

In Eq (5), *π*(*x*) means the distribution of the physical system. *A*(*x*) is the observed measurement. The calculation of the observation includes two integrals. ∫*A*(*x*)*π*(*x*)*dx* is calculated by using MC and *π*(*x*) is normalized using a density function. In the calculation of ∫*π*(*x*)*dx*, as normalization is not possible, Metropolis sampling is used to determine the Markov process. Thus, the full probability limit is proportional to the distribution. Metropolis sampling is a MC method used to sample from complex distributions, especially when direct sampling is difficult. Metropolis sampling is a stochastic process, which traverses the state space in a random way [28]. By proposing new states from a suggested distribution and accepting or rejecting them with a certain probability, the algorithm generates a series of samples based on the target distribution. After many iterations, the distribution of these samples will approximate the target distribution. In the process of IS, the MCMC model often suffers from local optima due to its excessive dependence on initial conditions [29]. This study replaces the subjective step of selecting initial parameters with MRF, thus constructing the MRF-MCMC image segmentation algorithm. The MRF-MCMC image segmentation algorithm flow is shown in Fig 3.

### 3.2 Adaptive image segmentation denoising algorithm based on MCMC sampling

**Fig 4:**
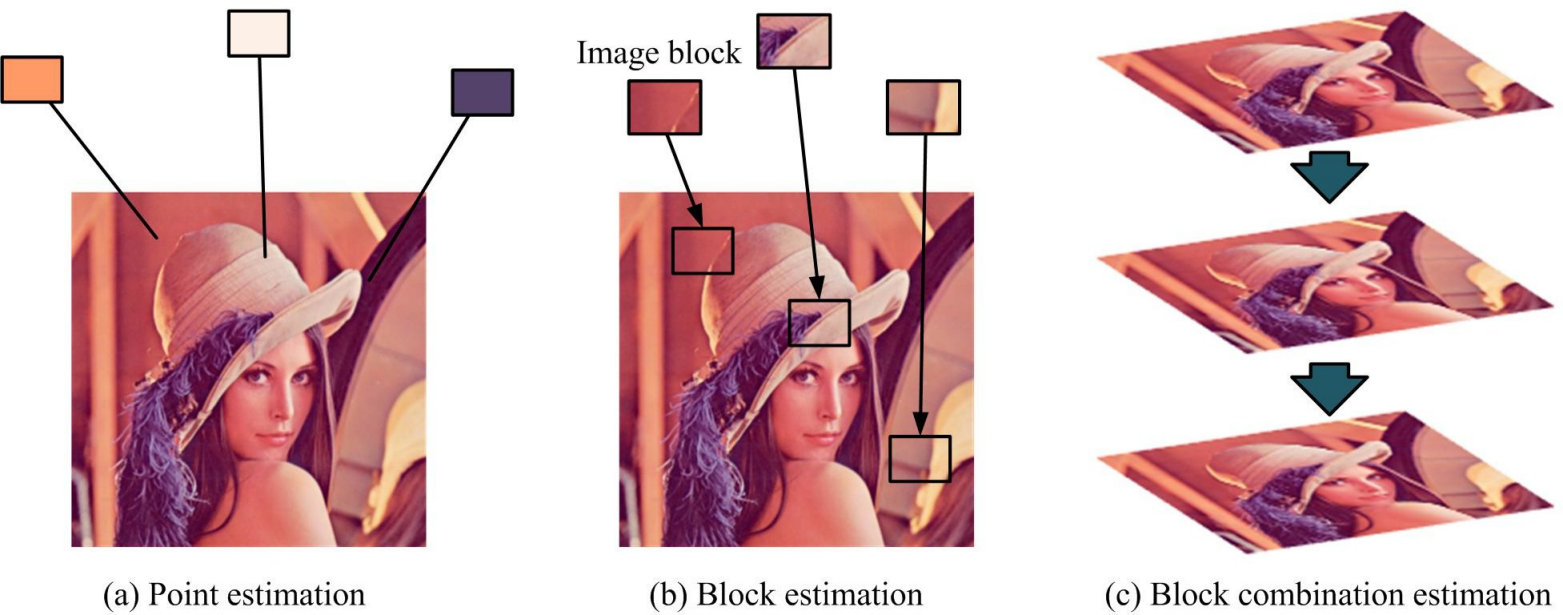
Three modes of non-local denoising. (a) Point estimation, (b) Block estimation, and (c) Block combination estimation.

Image segmentation refers to the process of dividing an image into different regions or sub regions, aiming to separate information such as targets, noise, and background in the image. To achieve better image segmentation results, it is necessary to denoise the segmented image and convert images containing noise or noise into purer sequences [30]. The non-local denoising method is commonly used at present. This method considers that the image is composed of many self-similarity structures, and noise denoising is achieved by averaging the structures [31]. The three modes of non-local denoising are shown in Fig 4.

In the non-local denoising algorithm, the mathematical model of Gaussian white noise is shown in Eq (6) [32].


z(x)=y(x)+n(x) (6)

In Eq (6), *z*(*x*) is the observed image. *y*(*x*) means the original image. *n*(*x*) represents noise. The expression for non-local average filtering is shown in Eq (7) [33].


$$NLz(x)=\sum_{y\in l}w(x,y)z(y)$$ (7)

In Eq (7), *w*(*x*,*y*) represents the weight coefficient, and its change reflects the similarity between pixels *x* and *y*, with $\sum_{y}w(x,y)=1$. The calculation of the weight coefficient is shown in Eq (8).


$$w(x,y)=\frac{1}{Z(x)}e^{-\frac{{\left\|z_{x}-z_{y}\right\|}_{2\sigma }^{2}}{h^{2}}}$$ (8)

In Eq (8), *Z*(*x*) expresses the normalization constant, and its calculation is shown in Eq (9).


$$Z(x)={\sum_{y}e}^{-\frac{{\left\|z_{x}-z_{y}\right\|}_{2\sigma }^{2}}{h^{2}}}$$ (9)

In Eq (9), *z*_*x*_ and *z*_*y*_ represent the neighboring grayscale value vectors of pixels *x* and *y*, respectively. ${\left\|z_{x}-z_{y}\right\|}_{2\sigma }^{2}$ is the Gaussian weighted Euclidean distance. *σ* means the standard deviation of the Gaussian kernel. *h* denotes a parameter that controls the degree of attenuation. Compared with traditional spatial denoising methods, non-local denoising introduces non-local ideas into the search of structures similar to the image itself. On the other hand, non-local denoising algorithms propose an image-based similarity measure that evaluates the success of denoising using similarity. If the accuracy of similarity estimation is insufficient, it will instead introduce non-similar pixels, thereby reducing the accuracy of the original image estimation. In traditional spatial denoising algorithms, grayscale and spatial distance are generally used to measure the similarity between pixels. However, in practical applications, these methods are not only computationally complex, but also susceptible to environmental influences and have poor robustness [34]. To study the combination of multiple statistical features based on segmented image blocks, an adaptive window similar block search algorithm with MCMC sampling is used to optimize non-local denoising algorithms. During the operation of this algorithm, the size *d* of similar blocks is first given, and MCMC is used to search for the neighborhood block similarity sequence Ω^*d*^(*S*_*j*_) of pixel point *s*_*j*_ in the graph. Then the expected $E_{p}^{d}(s_{j})$ of the similar block sequence is calculated. The average similar block *E*^*d*^(*s*_*j*_) of the neighboring blocks of multiple pixel points *s*_*j*_ is obtained. The variance *V*^*d*^(*s*_*j*_) of *E*^*d*^(*s*_*j*_) is calculated. Finally, it needs to determine whether it is necessary to adjust the size of similar blocks based on the variance *V*^*d*^(*s*_*j*_). In the adaptive window-based similarity block search algorithm, the neighborhood of MCMC sampling points needs to reflect both strong locality and satisfy global sampling. The sampling process can be understood as a local global sampling, so its distribution definition is shown in Eq (10).


$$Q(s_{jk}^{'}|s_{jk-1})=\frac{1}{\sqrt{2\pi \sigma_{s}}}exp[-(\frac{{\left(s_{kj}^{'}-s_{jk-1}\right)}^{2}}{2\sigma_{s}})]$$ (10)

In Eq (10), *σ*_*s*_ means the spatial variance of $Q(s_{jk}^{'}|s_{jk-1})$. $s_{jk}^{'}$ expresses the sampling point. *s*_*jk*_ stands for the center point. To determine whether the sampling point is a point in $\Omega_{p}^{d}(s_{j})$, the acceptance probability function of the sampling point is established, as shown in Eq (11).


$$\alpha (s_{jk}^{'}|s_{jk-1})=min\{1,\frac{\varphi (s_{jk}^{'}|s_{0})}{\varphi (s_{jk-1}^{'}|s_{0})}\}$$ (11)

In Eq (11), *s*_0_ means the estimation point. $\varphi (s_{jk}^{'}|s_{0})$ is an objective evaluation function used to evaluate the similarity between sampling points and estimation points. To facilitate the evaluation of the similarity between the two, a similarity evaluation function is defined as shown in Eq (12).


$$\varphi (s_{jk}^{'}|s_{0})=\prod_{i}exp[-\frac{{\left(F^{\rho_{s_{jk}^{'}}}(i)-F^{\rho_{s_{jk}}}(i)\right)}^{2}}{C}]$$ (12)

In Eq (12), $\rho_{s_{jk}^{'}}$ is the neighborhood block of $s_{jk}^{'}$. $\rho_{s_{j0}}$ means the neighborhood block of *s*_*jk*_. *F*(*i*) expresses the pixel value of the pixel point *i*. *C* represents a constant. The second step of the algorithm adjusts the size of similar blocks by averaging the consistency of the estimated set of similar blocks. This study selects the mean and variance of similar block set estimation as statistics to evaluate consistency. When there are enough similar blocks, the variance between the average similar blocks is very small, indicating consistency between them. At this point, their size can be increased. When there are not enough similar blocks, there are significant differences between them, indicating that they do not have consistency and need to be reduced in size. The algorithm ultimately needs to adjust the size of similar blocks to obtain the optimal set of similar blocks and blocks. The calculation of the optimal similar block size is shown in Eq (13).


$$d=max\{d_{i}|V({\left\{E_{p}^{d_{i}}(s_{j})\right\}}_{p=1}^{N})<T\}$$ (13)

**Fig 5:**
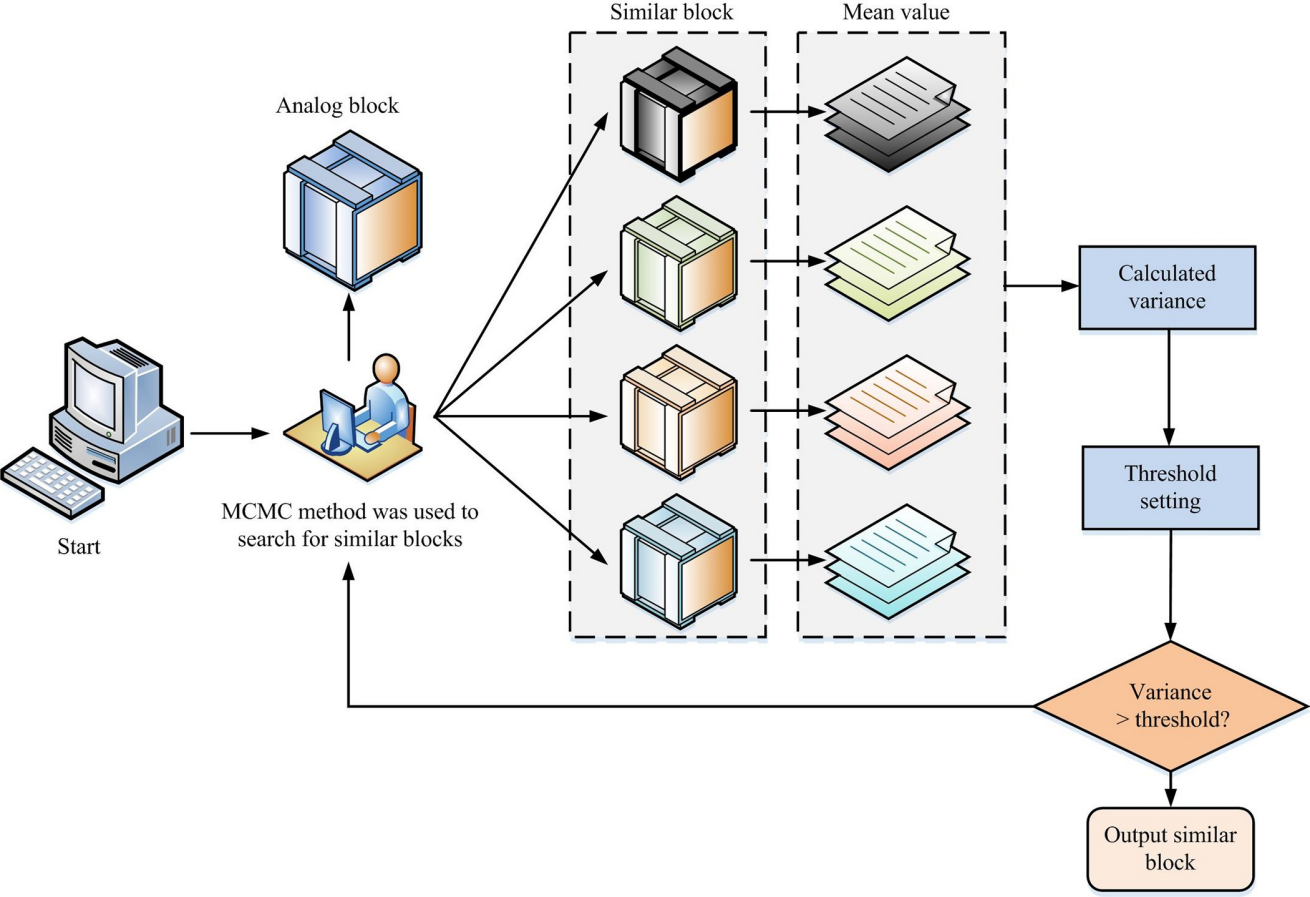
Adaptive window similar block search.

In Eq (13), $T=\frac{1}{\sigma^{2}}$ represents the threshold and *σ*^2^ is the noise variance. The search for similar blocks in adaptive variable size windows is shown in Fig 5.

When similar blocks are selected, denoising treatment needs to be taken. Currently, a large number of processing methods use image block similarity as a weight to estimate the grayscale values of pixels. However, in practical applications, although these methods have achieved certain denoising effects, they have caused damage to the structure and details of the image itself [35]. The adaptive denoising algorithm based on MCMC sampling proposes in this study. The algorithm consists of two stages: the first stage is similar block search for adaptive size windows, and the second stage is a bidirectional non-local denoising algorithm. In the first stage, the study searches for multiple sets of similar matching blocks through MCMC sampling until obtaining the target number of similar blocks. In the second stage, the traditional bidirectional non-local algorithm is modified by utilizing the bidirectional similarity structure of the selected similar block set images. The estimation of the set of noiseless similar blocks obtained through correction not only simplifies the complex calculation, but also ensures good denoising performance.

## 4 Application testing of MRF-MCMC in IS

To ensure that the experimental environment does not cause errors in the results, the same computer equipment was used for simulation testing in this experiment. Information about the experimental environment is given in Table 1.

**Table 1:** Experimental environment information.

Name	Configuration
Video card	GTX 1080ti
CPU	Inter(R)Core(TM)i5-7200U
Gpu-accelerated library	CUDA 10.0
Memory	64 GB
Operating system	Windows 10
Platform	MATLAB R2014a

**Fig 6:**
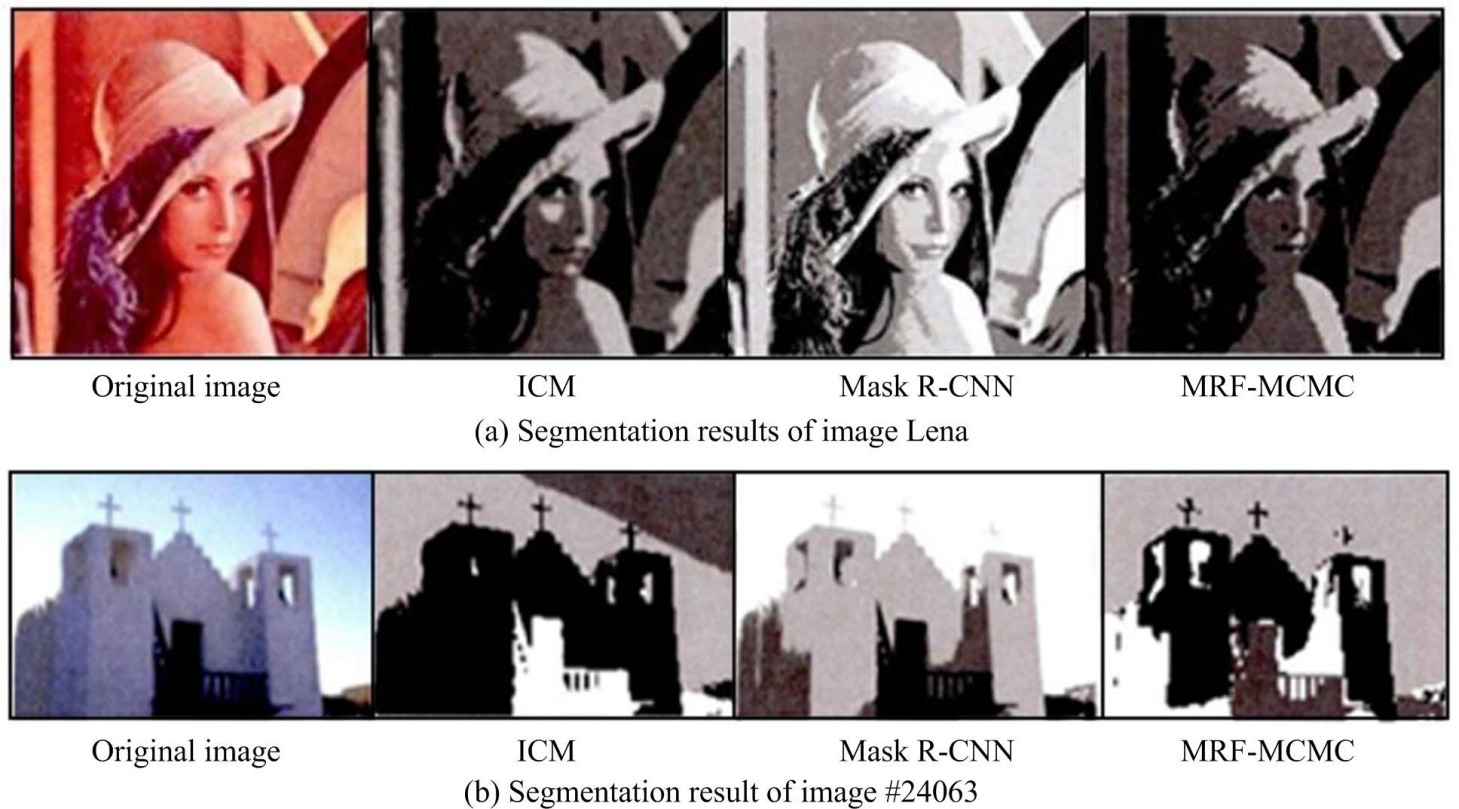
Comparison of image segmentation results. (a) Segmentation results of image Lena and (b) Segmentation result of image #24063.

To verify the segmentation effect of the MRF-MCMC segmentation algorithm proposed in the study, Lena original images and Berkeley segmentation datasets commonly used in the field of image segmentation were selected for segmentation experiments. A random image (# 24063 image) was selected from the experimental results and analyzed together with the Lean original images. The comparison algorithm used the iterative conditional modes (ICM) algorithm commonly used in the image segmentation field to find the maximum number of conditional probability and the more advanced mask region based Convolutional neural network (Mask R-CNN) [36]. The result is shown in Fig 6.

From Fig 6, the algorithm proposed in this study had better segmentation performance and accuracy. In the segmentation experiment of Lena image in Fig 6(A), MRF-MCMC segmentation was more detailed with fewer misclassifications. Overall, MRF-MCMC outperformed Mask R-CNN and Mask R-CNN outperformed ICM in overall performance. In the segmentation experiment of building images in Fig 6(B), MRF-MCMC performed more accurate segmentation on details such as windows and stairs, with better results than the other two types of segmentation algorithms. The running time of different algorithms is shown in Table 2.

**Table 2:** Comparison of iteration times of different algorithms.

Category	Run time (s)		
	ICM	Mask R-CNN	MRF-MCMC
Image	19.898	20.655	17.874
Lena			
Berkeley	9.109	10.733	8.762
Segmentation			
dataset			

**Fig 7:**
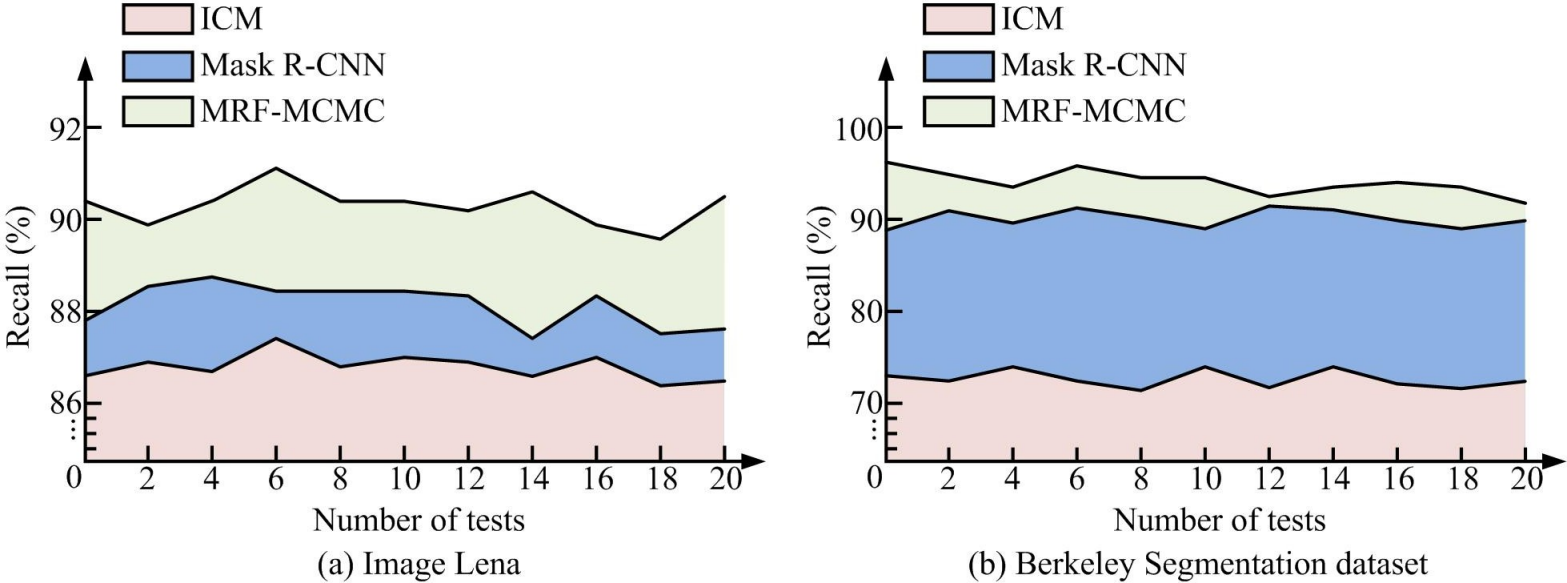
Comparison of recall rates. (a) Image Lena and (b) Berkeley Segmentation dataset.

From Table 2, in Lena image and # 24063 image segmentation, the MRF-MCMC algorithm had the shortest iteration time, followed by the ICM, and the Mask R-CNN had the longest iteration time. The algorithm proposed in this study was verified to have better segmentation efficiency and less running time. Compared to the Mask R-CNN algorithm, the iteration time of MRF-MCMC in Lena image segmentation had been reduced by 13.46%, and the iteration time in # 24063 image segmentation had been reduced by 18.36%. Compared to the ICM algorithm, the iteration time of MRF-MCMC in Lena image segmentation had been reduced by 10.17%, and the iteration time in # 24063 image segmentation had been reduced by 3.81%. The outcomes validated the effectiveness of the improvement method proposed in this study. To evaluate the performance of the MRF-MCMC image segmentation algorithm more accurately, the study measured the performance of the image segmentation algorithm in terms of three metrics, namely, recall, precision, and DICE coefficient. Among them, the recall was the proportion of correctly categorized pixels among all the pixels that should be segmented out. The recall measured the ability of the algorithm to recognize the pixels that should be segmented out. If the recall was higher, it meant that the possibility of missed detection was less. The precision measured the proportion of pixels segmented by the algorithm that really should have been segmented. If the precision was higher, it meant that the likelihood of false detection was lower. The DICE coefficient was a measure of the similarity between two samples. In image segmentation, it was often used to measure the similarity between the region segmented by the algorithm and the region that should actually be segmented. The DICE coefficient was a reconciled average of the precision rate and the recall rate, which balanced the effects of the two. If the DICE coefficient was higher, it meant that the algorithm was more effective in segmentation. The comparison results of recall rates were shown in Fig 7.

**Fig 8:**
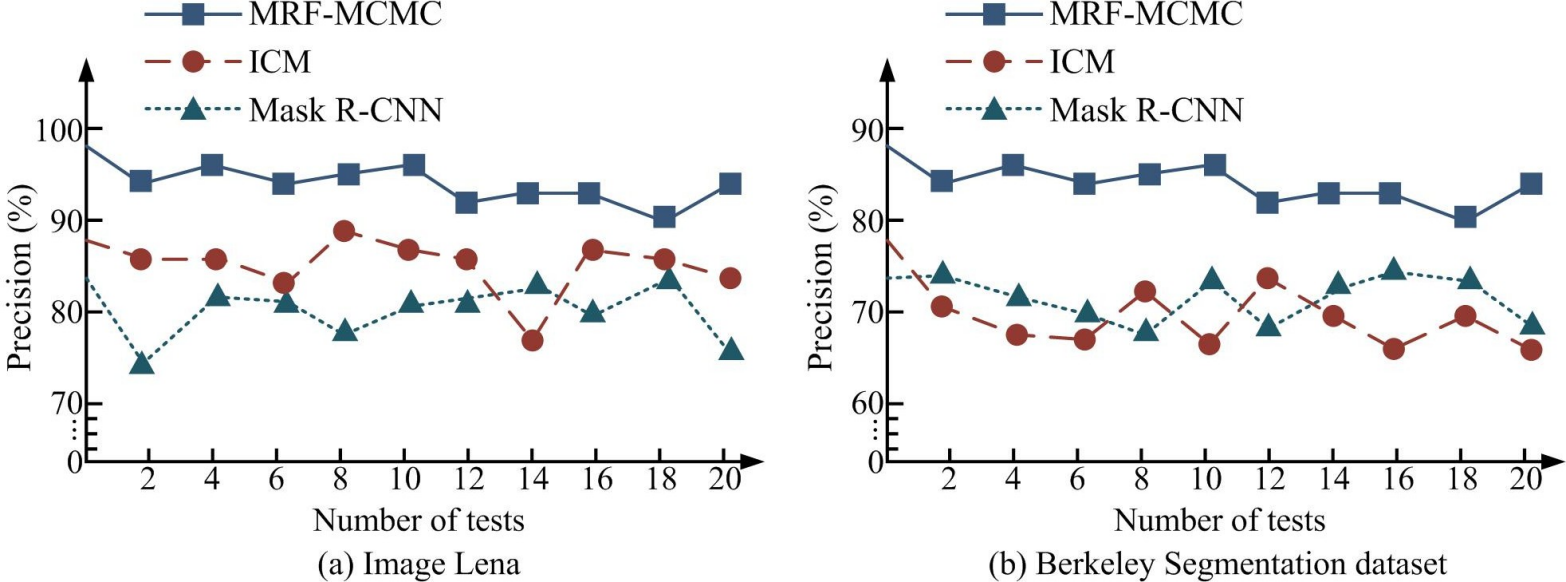
Comparison of algorithm accuracy. (a) Image Lena and (b) Berkeley Segmentation dataset.

Fig 7 shows the recall comparison results for the different algorithms. Fig 7(A) is the comparison of recall between different segmentation algorithms in Lena images. From the graph, in 20 experiments, the MRF-MCMC algorithm had the best recall performance, significantly higher than the other two types of segmentation algorithms. The recall of Mask R-CNN algorithm was slightly higher than that of ICM algorithm. Fig 7(B) shows the comparison of recall among different segmentation algorithms in Berkeley segmentation dataset. From the graph, in 20 experiments, the MRF-MCMC algorithm had the best recall performance, slightly higher than the Mask R-CNN algorithm. The recall rate of MRF-MCMC and Mask R-CNN algorithm was significantly higher than that of ICM algorithm. The experimental results denoted that the MRF-MCMC algorithm outperformed the Mask R-CNN and ICM algorithms in correctly segmenting images. The accuracy of image segmentation was compared using three algorithms, and the results are shown in Fig 8.

**Fig 9:**
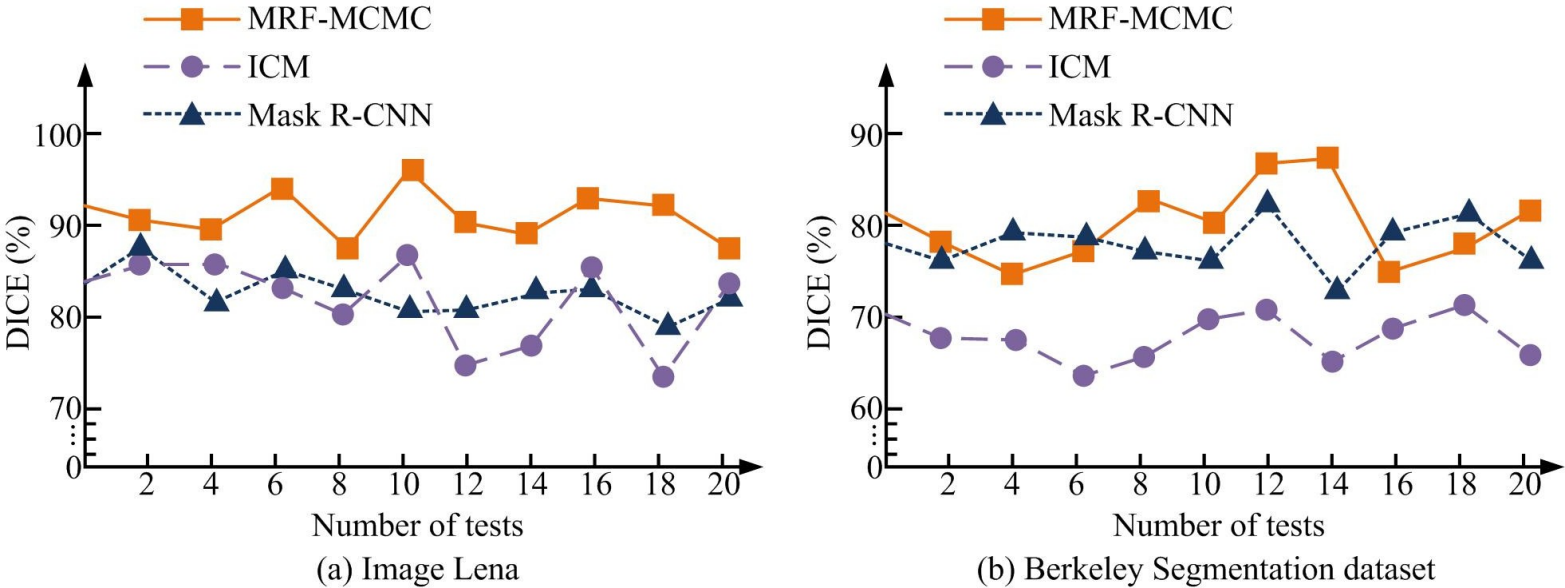
Comparison of algorithm dicesimilarity coefficient. (a) Image Lena and (b) Berkeley Segmentation dataset.

Fig 8 shows the results of the accuracy comparison for the different algorithms. Accuracy means the proportion of the intersection and union of the amount of rightly segmented pixels and the true value in image segmentation. Fig 8(A) shows the accuracy comparison of different segmentation algorithms in Lena images. From the graph, in 20 experiments, the accuracy of the MRF-MCMC algorithm was higher than the other two types of algorithms, with an accuracy of over 90%. The accuracy of ICM was lower than that of Mask R-CNN algorithm in the 14th experiment, and higher than Mask R-CNN algorithm in other experiments. Fig 8(B) shows the accuracy comparison of different segmentation algorithms in Berkeley segmentation dataset. From the graph, in 20 experiments, the accuracy of the MRF-MCMC algorithm was higher than the other two algorithms, with an accuracy rate of over 80. The accuracy of ICM and Mask R-CNN was similar. In the first six experiments, the accuracy of Mask R-CNN algorithm was slightly higher, while in the last six experiments, the accuracy of ICM algorithm was slightly higher. The experimental results showed that the accuracy of MRF-MCMC algorithm in image segmentation was better than that of ICM and MCMC algorithms. The DICE coefficients of three algorithms were compared in IS, and the results were shown in Fig 9.

Fig 9 shows the DICE coefficients of different algorithms. In the evaluation of image segmentation effectiveness, the proportion of twice the intersection of the segmentation result and the true value to the total of the two were calculated by the DICE coefficient. Fig 9(A) showed the comparison of DICE coefficients between different segmentation algorithms in Lena images. From the graph, in 20 experiments, the DICE coefficient of MRF-MCMC algorithm was higher than the other two algorithms, with a DICE coefficient of over 85%. The DICE coefficients of ICM and Mask R-CNN algorithms were close and there was no significant difference. Fig 9(B) showed the comparison of DICE coefficients between different segmentation algorithms in Berkeley segmentation dataset. From the graph, in 20 experiments, the DICE coefficients of MRF-MCMC and Mask R-CNN algorithms were significantly higher than those of ICM algorithm. The DICE coefficients of Mask R-CNN were higher than those of MRF-MCMC algorithm in the 3rd, 4th, 6th, 16th, 17th, and 18th experiments, and lower than MRF-MCMC algorithm in other experiments. The experimental results indicated that in Lena images, the MRF-MCMC algorithm significantly outperformed the ICM and Mask R-CNN algorithms in segmenting image DICE coefficients. Although the DICE coefficient in Berkeley segmentation dataset was better than the ICM algorithm, there was no significant difference compared to the MCMC algorithm. It calculated the average values of accuracy, recall, and DICE coefficients in the experiment, and the specific data results were obtained as shown in Table 3.

**Table 3:** The average of accuracy rate, recall rate and dicesimilarity coefficient.

Category	Accuracy (%)			Recall (%)			DICE (%)		
	ICM	Mask R-CNN	MRF-MCMC	ICM	Mask R-CNN	MRF-MCMC	ICM	Mask R-CNN	MRF-MCMC
Image	86.95	79.66	94.26	87.29	87.98	90.72	84.89	85.88	92.39
Lena									
Berkeley	70.19	73.03	82.77	72.23	91.84	94.24	69.84	78.71	80.72
Segmentation									
dataset									

As shown in Table 3, the average segmentation accuracy of MRF-MCMC in Lena image and Berkeley segmentation dataset was 94.26% and 82.77%, respectively. The average recall rate of MRF-MCMC in Lena images was 90.72%, and the average segmentation accuracy in Berkeley Segmentation dataset was 94.24%. The average DICE coefficient of MRF-MCMC in Lena image and Berkeley segmentation dataset was 92.39% and 80.72%, respectively. From the data in the table, the MRF-MCMC algorithm performed best in all indicators in image segmentation experiments, verifying the effectiveness of this study.

**Fig 10:**
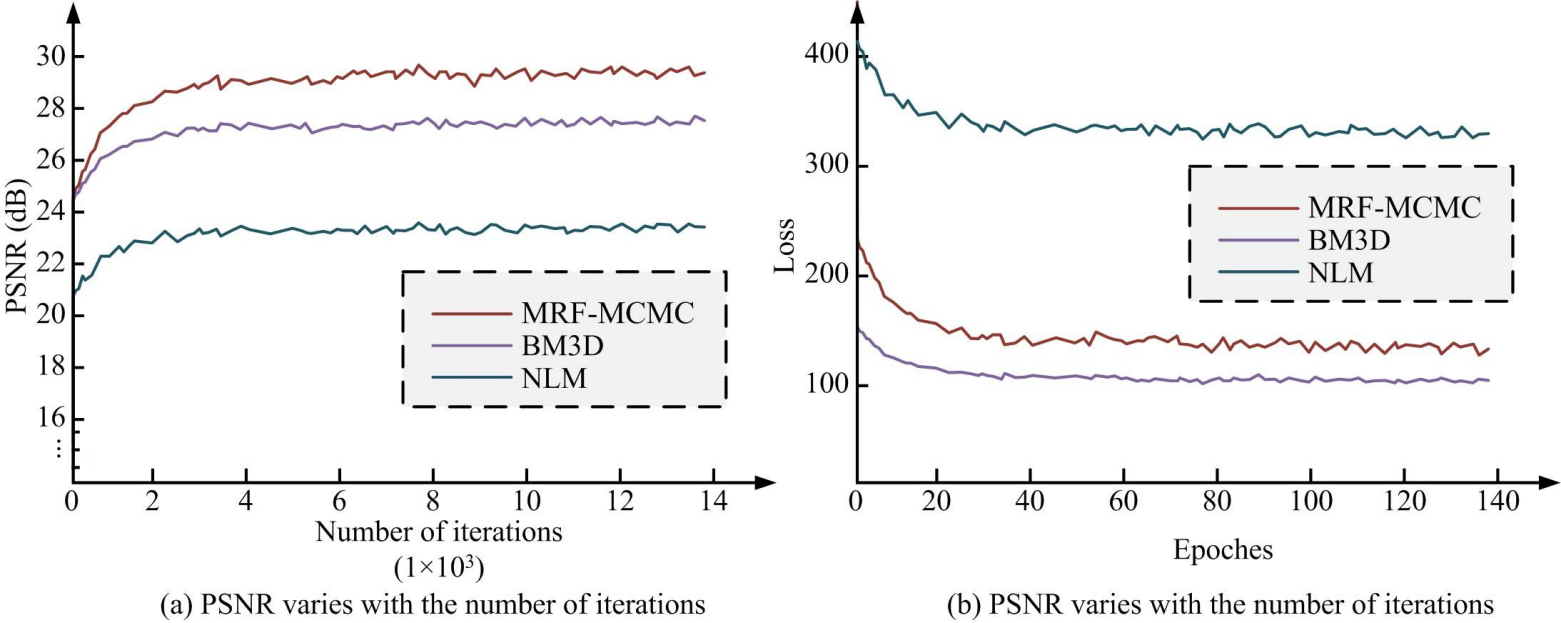
Comparison of training results between peak signal-to-noise ratio and structural similarity. (a) PSNR varies with the number of iterations and (b) PSNR varies with the number of iterations.

In the MRF-MCMC algorithm model, an adaptive segmentation image denoising algorithm was proposed by combining MCMC sampling. To verify the denoising effect of the MRF-MCMC algorithm, experiments were conducted using the Tensorflow deep learning framework. Common image denoising algorithms were compared with Block matching and 3D filtering (BM3D) algorithm and Non-Local Mean (NLM) algorithm [37]. Performance comparison metrics included Peak Signal-to-Noise Ratio (PSNR) and Structural Similarity (SSIM). Among them, PSNR was one of the most commonly used metrics to evaluate image quality. It compared the difference between original and denoised images, and quantified the sum of squares of errors due to noise or distortion. The higher the PSNR value, the better the quality of the image, i.e., the better the denoising effect. SSIM is another metric for evaluating the quality of an image, which takes into account the brightness, contrast, and structural information of the image. SSIM metric evaluated the image quality by comparing the difference between original and denoised images in terms of structure, brightness, and contrast. The value of SSIM was between -1 and 1. 1 meant that the two images were exactly the same and the closer to 1 meant the better the denoising effect. It got the training results of PSNR and loss function, as shown in Fig 10.

Fig 10(A) shows a comparison of the training results of PSNR. The initial PSNR of the MRF-MCMC algorithm was close to that of the BM3D algorithm and higher than that of the NLM algorithm. The initial PSNR of the MRF-MCMC, BM3D, and NLM algorithms was 24.85dB, 24.79dB, and 21.09dB, respectively. Fig 10(B) shows the comparison of loss function training. MRF-MCMC algorithm tended to converge after 30 rounds of training, while BM3D and NLM tended to converge after 40 rounds of training. Compared to the other two types of algorithms, the MRF-MCMC algorithm had faster convergence speed and lower loss values. The experimental results verified the effectiveness of MRF-MCMC.

**Fig 11:**
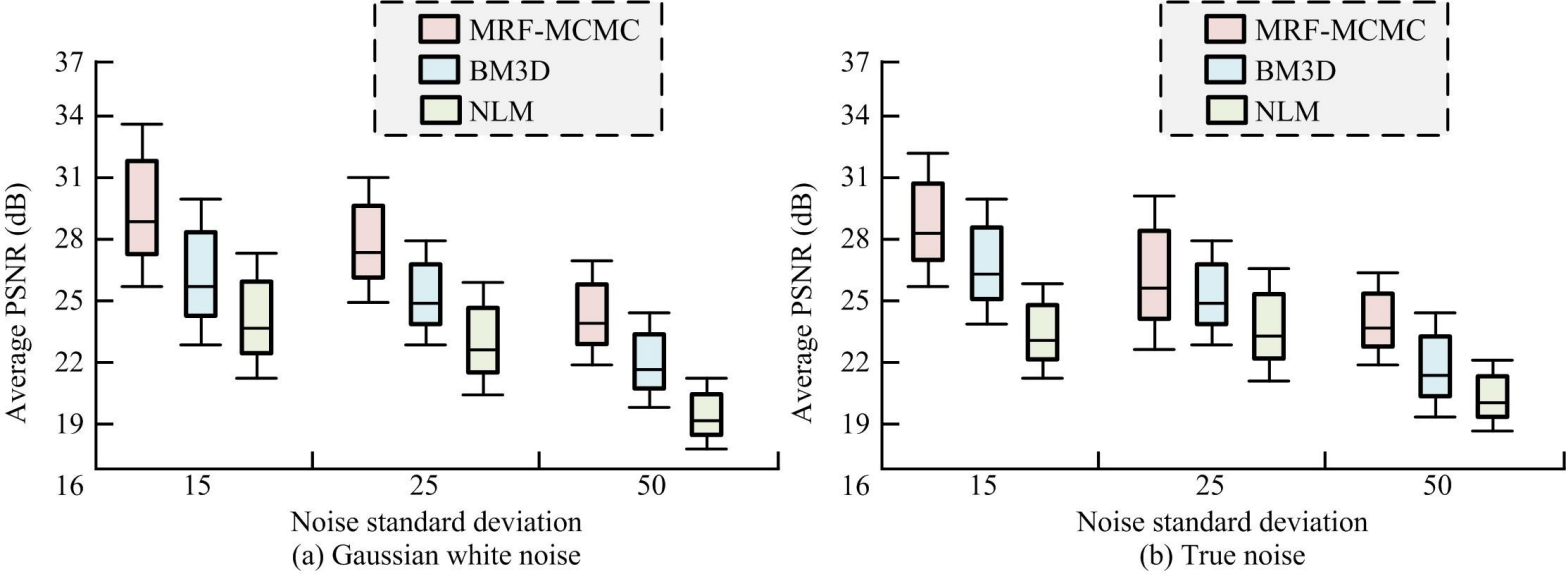
Average peak signal-to-noise ratio of different algorithms on the test set. (a) Gaussian white noise and (b) True noise.

To compare the PSNR and SSIM performance of the algorithm, the study added additive Gaussian white noise with and true noise standard deviation of 15, 25, and 50 to the original test image, respectively. The real noise chosen for the study is sensor noise. Sensor noise is usually generated during the image capture phase due to the physical properties of the image sensor and external environmental conditions (e.g., temperature and illumination). Real noise helps to more accurately reflect the performance of the algorithm in real application scenarios, thus providing valuable insights for the development of image denoising techniques. It drew the average PSNR box plot of different algorithms on the test set, as shown in Fig 11.

**Fig 12:**
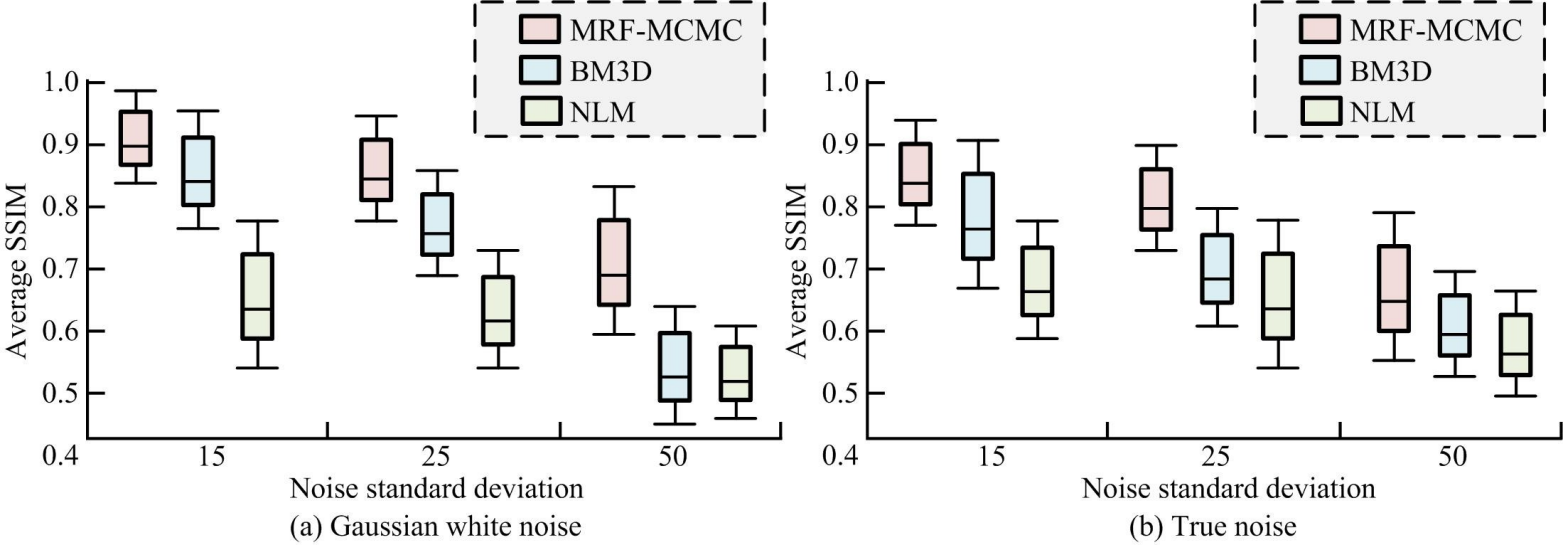
Average structural similarity of different algorithms on the test set. (a) Gaussian white noise and (b) True noise.

Fig 11(A) shows the average PSNR performance of the algorithm under Gaussian white noise. From the figure, MRF-MCMC had the highest PSNR values at noise standard deviations of 15, 25, and 50. When the standard deviation was 15, 25, and 50, the average PSNR value was 30.03dB, 28.24dB, and 24.73dB, respectively. Compared to BM3D, the average PSNR of MRF-MCMC increased by 13.45%, 11.54%, and 9.19% at noise standard deviations of 15, 25, and 50, respectively. Compared to NLM, the average PSNR of MRF-MCMC increased by 21.94%, 21.74%, and 23.68% at noise standard deviations of 15, 25, and 50, respectively. Fig 11(B) shows the average PSNR performance of the algorithm under real noise. From the figure, MRF-MCMC had the highest PSNR value at different standard deviations, indicating the best image denoising. Compared to compared to BM3D, MRF-MCMC improved the average PSNR by 8.89%, 9.07% and 8.23% at noise standard deviation of 15, 25 and 50, respectively. Compared to NLM, MRF-MCMC improved the average PSNR by 34.53%, 20.15% and 39.41% at noise standard deviation of 15, 25 and 50, respectively. The experimental results validated the effectiveness of the optimization method proposed in the study. It drew the average SSIM box plots of different algorithms on the test set, as shown in Fig 12.

Fig 12(A) shows the average SSIM performance of the algorithm under Gaussian white noise. From the figure, the SSIM values of MRF-MCMC were the highest at noise standard deviations of 15, 25, and 50. When the standard deviation was 15, 25, and 50, the average SSIM value was 0.91, 0.88, and 0.72, respectively. Compared to BM3D, the average SSIM of MRF-MCMC increased by 7.12%, 12.83%, and 18.29% at noise standard deviations of 15, 25, and 50, respectively. Compared to NLM, the average SSIM of MRF-MCMC increased by 43.23%, 45.19%, and 41.18% at noise standard deviations of 15, 25, and 50, respectively. Fig 12(B) shows the average SSIM performance of the algorithm under real noise. From the figure, MRF-MCMC had the highest SSIM values at noise standard deviation of 15, 25, and 50, which indicated better image structure similarity before and after denoising. Compared to BM3D, MRF-MCMC has improved the average SSIM by 11.23%, 18.27% and 21.09% at noise standard deviation of 15, 25 and 50, respectively. Compared to NLM, MRF-MCMC improved the average SSIM by 39.72%, 37.84% and 46.32% at noise standard deviation of 15, 25, and 50, respectively. Overall, MRF-MCMC had the best denoising performance, followed by BM3D, while NLM had relatively poor denoising performance. The results verified that the proposed adaptive segmentation image denoising algorithm based on MCMC sampling had good denoising ability and good application value.

## 5 Discussion

Image segmentation, as a core component of computer vision and image processing, plays a vital role in various applications such as medical image analysis, environmental monitoring, urban planning, etc. In this context, the research proposed MRF-MCMC-based image segmentation algorithm brings new possibilities in this field. Studying the existing MRF basis, the algorithm significantly reduces the dependence of image segmentation on a priori knowledge, thus effectively dealing with problems such as imaging noise. In addition, by utilizing MCMC, the study successfully transforms the complex integration problem into a probability distribution expectation, enabling estimation by sampling. This innovative approach avoids the extensive manual intervention required for conventional image segmentation. To solve the problem that the MCMC model is overly dependent on the initial conditions and prone to fall into local optimal solutions, the study introduces the non-local idea to further optimize the similar structure search in the image itself. In addition, the study also utilizes multiple statistical properties of the image and combines the adaptive window similarity block search algorithm sampled by MCMC to optimize the non-local denoising algorithm, which results in a purer image and improved image segmentation. In the experimental results, the proposed algorithm of the study performs well in several key metrics. Overall, the research opens the way to a new method of image segmentation based on MRF-MCMC, which provides new possibilities for dealing with complex image problems, although there is still a lot of work to be done, the research is confident in the future of this method.

## 6 Conclusion

This research presented an innovative image segmentation algorithm that combined MRF and MCMC. This approach significantly reduced the dependence on a priori knowledge for image segmentation and effectively handled issues such as imaging noise. In addition, the algorithm introduced the non-local idea to optimize the search of the image’s own similar structure, which made the image purer and thus improved the image segmentation. In the experimental results, MRF-MCMC performed better than the other two algorithms in the segmentation of Lena images and building images. The average recall, precision, and DICE coefficient of MRF-MCMC algorithm were 92.48, 88.52, and 86.56, respectively. And all of the metrics were better than ICM and MCMC algorithms. In addition, the denoising algorithm proposed in this study converged faster, had lower loss during training, and had a higher average PSNR and SSIM than the widely used BM3D and NLM denoising algorithms on the test set. This research provided a more efficient method for image segmentation and provides new guiding ideas in the field of digital image processing. However, due to the limitation of equipment conditions, the study failed to conduct large-scale experiments, so the experimental results could only provide some references for optimizing image segmentation algorithms. The main research direction in the future will be to improve the applicability of MRF-MCMC and verify its application effect on large samples.
